# Compensatory adaptation and diversification subsequent to evolutionary rescue in a model adaptive radiation

**DOI:** 10.1002/ece3.7792

**Published:** 2021-06-16

**Authors:** Dong‐Hao Zhou, Quan‐Guo Zhang

**Affiliations:** ^1^ State Key Laboratory of Earth Surface Processes and Resource Ecology and MOE Key Laboratory for Biodiversity Science and Ecological Engineering, College of Life Sciences Beijing Normal University Beijing China

**Keywords:** adaptive radiation, environmental stress, experimental evolution, fitness landscape, *Pseudomonas fluorescens*

## Abstract

Biological populations may survive lethal environmental stress through evolutionary rescue. The rescued populations typically suffer a reduction in growth performance and harbor very low genetic diversity compared with their parental populations. The present study addresses how population size and within‐population diversity may recover through compensatory evolution, using the experimental adaptive radiation of bacterium *Pseudomonas* *fluorescens*. We exposed bacterial populations to an antibiotic treatment and then imposed a one‐individual‐size population bottleneck on those surviving the antibiotic stress. During the subsequent compensatory evolution, population size increased and leveled off very rapidly. The increase of diversity was of slower paces and persisted longer. In the very early stage of compensatory evolution, populations of large sizes had a greater chance to diversify; however, this productivity–diversification relationship was not observed in later stages. Population size and diversity from the end of the compensatory evolution was not contingent on initial population growth performance. We discussed the possibility that our results be explained by the emergence of a “holey” fitness landscape under the antibiotic stress.

## Graphical abstract

Bacterial populations lost diversity and showed reduced growth performance after an evolutionary rescue from environmental stress. During the subsequent compensatory adaptation, improvement of growth performance preceded ecological diversification, and the final population size and diversity were not contingent on initial population growth performance. The mode and pace of adaptation and diversification may be explained by a holey fitness landscape under environmental stress.

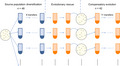

## 1 INTRODUCTION

Environmental changes have threatened the persistence of many local biological populations and also caused a decline of biodiversity on the global scale. Processes that may promote population survival in degraded habitats have been studied extensively, among which is evolutionary rescue (Alexander et al., 2014; Bell, 2017; Bell & Collins, 2008; Bell & Gonzalez, 2009; Carlson et al., 2014; Gomulkiewicz & Holt, 1995; Gonzalez et al., 2013; Mueller et al., 2020). When a population is exposed to lethal environmental conditions, genotypes resistant to the environmental stress may increase in frequency sufficiently fast to recover population growth. Greater supply of genetic variation (resulting from, e.g., larger population size and more frequent immigration) and weaker selection (associated with milder environmental stress) have been suggested as conditions that favor the occurrence of evolutionary rescue (reviewed in Bell, 2017; Carlson et al., 2014). Studies with both laboratory and filed populations suggest that evolutionary rescue could be prevalent, particularly in organisms with large population sizes and short‐generation times, and may have profound consequences for the maintenance of biodiversity and ecosystem functioning under future environmental change scenarios (Bell et al., 2019; Bell & Gonzalez, 2009; Lohbeck et al., 2012; Ramsayer et al., 2013; Rinkevich, 2019; Sunday et al., 2014).

While evolutionary rescue shows promise for enhancing population persistence and biodiversity maintenance, it is not without costs. In particular, evolutionarily rescued populations should typically have poorer growth performance as resistance to environmental stress usually incurs fitness costs (Andersson & Hughes, 2010, 2011; Björkman et al., 1998; Gassmann et al., 2009; Melnyk et al., 2014; Vila‐Aiub et al., 2009). They may also harbor very low genetic diversity due to selective sweeps (Orr & Unckless, 2014; Osmond & Coop, 2020; Wilson et al., 2017). Hence, evolutionary rescue may actually leave legacies that jeopardize ecological functions and the potential for future adaptation. Knowledge about how those negative “side‐effects” of evolutionary rescue could be offset by further adaptive evolution under the stressful environments (namely compensatory adaptation; (Maisnier‐Patin & Andersson, 2004; Meftahi et al., 2016; Nagaev et al., 2001; Perron et al., 2010)) could have crucial implications for practices of conservation biology and ecosystem restoration.

Different local populations rescued from a same environmental stress may be founded with different genotypes that bear distinct resistance mutations, and those genotypes likely vary in their growth performance (Collins & de Meaux, 2009; Killeen et al., 2017; Lindsey et al., 2013; Perron et al., 2008). Two hypotheses may be formalized for the tempo and mode of the recovery of growth performance and within‐population ecological diversity. First, general population genetics and competition theory predict that populations with better initial growth performance will show faster compensatory evolution. This is because larger population sizes resulting from improved growth would increase mutational supply for further evolutionary changes (Handel & Rozen, 2009; Payne & Wagner, 2018; de Visser & Rozen, 2005); and greater population productivity may also strengthen diversifying selection forces that promote niche differentiation (Buckling & Rainey, 2002; Meyer & Kassen, 2007; Zhang et al., 2018). Therefore, improvement of growth performance should precede ecological diversification during the course of compensatory evolution. Second, a “rugged” fitness landscape paradigm for understanding population diversification would predict that populations with poorer initial growth performance (fitness) have a larger chance to show ecological diversification, as it is harder for a better‐adapted population (near a fitness peak) to cross a fitness valley to explore alternative fitness peaks (Bittihn & Tsimring, 2017; Buckling et al., 2003; Hayashi et al., 2006; Weissman et al., 2009). If this is true, there will be prolonged improvement of growth performance after ecological diversification.

Here, we address the recovery of population size (environmental carrying capacity) and ecological diversity (diversity relevant to niche differentiation) during compensatory adaptation, using a model microbial adaptive radiation system. Cultures of the bacterium *Pseudomonas* *fluorescens* SBW25 (Rainey & Bailey, 1996), when grown in static microcosms, can diversify rapidly into a number of types distinguishable by colony morphology. Those morphotypes fall into three ecotypes occupying different spatial niches: smooth morph types (SM) at the broth phase, wrinkly spreaders (WS) forming a biofilm at the air‐broth interface, and fuzzy spreaders (FS) at the bottom of microcosms. Coexistence between the three ecotypes is stable, and morphotypes within a certain ecotype may coexist stably or neutrally (Fukami et al., 2007; Rainey & Travisano, 1998; Zhang et al., 2009).

## 2 MATERIALS AND METHODS

### 2.1 Bacterial strain and culture conditions

*P. fluorescens* SBW25 was used in this study (Rainey & Bailey, 1996). Bacteria were grown at 28°C in static microcosms, 50 ml centrifuges with loosened lids each of which contained 5 ml of M9KB medium. For every 2 days, 50 μl of each culture was transferred into a fresh microcosm.

### 2.2 The experiments

**FIGURE 1:**
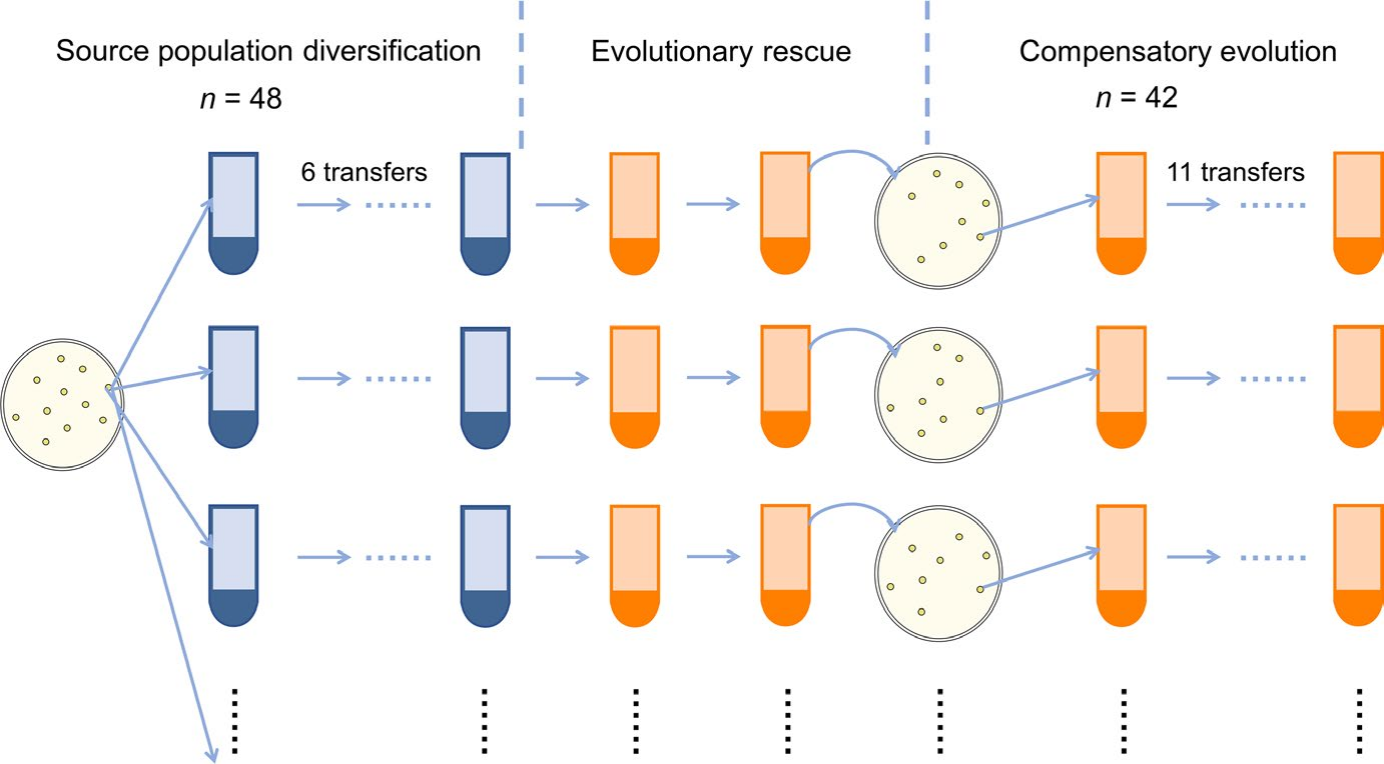
Schematic representation of the experimental design. Blue tubes show antibiotic‐free environment, and orange tubes show stressful environment with the antibiotic

Figure 1 graphically illustrated the experimental design. First, 48 replicate source populations founded with a single ancestral bacterial isolate evolved in antibiotic‐free medium for six transfers, a period allowing within‐population diversity to approach an equilibrium (Fukami et al., 2007; Tan et al., 2013). Population density was measured by spreading dilutions of cultures onto agar plates and counting colony‐forming units (CFUs); and morphotype richness of each population was estimated based on approximately 100 randomly chosen colonies (Buckling et al., 2000; Kassen et al., 2000). Second, the 48 source populations were transferred into a stressful environment, M9KB medium supplemented with 14 μg/ml kanamycin (approximately twofold the minimum inhibitory concentration). Forty‐two out of 48 populations survived the antibiotic treatment. Each of the surviving populations was then imposed upon a one‐individual‐size population bottleneck, by spreading dilutions of cultures onto agar plates and choosing a colony from the most numerically dominant morphotype to inoculate a new microcosm. This experimental bottleneck reduced the diversity of each population to a same, very low, level, to ensure that diversity during the compensatory evolution would result from new diversification events, but not standing variation. Third, the 42 populations were allowed to evolve in antibiotic‐present medium for another eleven transfers, with population density and diversity measured regularly. A variety of SM and WS, but not FS, morphotypes were observed in our experiment (more details in Table S1).

### 2.3 Statistical analysis

Data analyses were performed using R (R Core Team, 2020). Population density and morphotype richness data were log‐transformed before analysis. Each of our populations consisted of only one or two ecotypes. Therefore, we analyzed ecological diversification state as binary data, and specifically, 1‐ and 2‐ecotype populations were given scores of “0” and “1” (ecologically nondiversified and diversified), respectively. Changes in population density and morphotype richness over time during compensatory evolution were analyzed using linear mixed‐effect models (“lmer” function provided by R package “lme4”), where time was included as a continuous explanatory variable, and population ID as a random factor. Ecological diversification state (binary data) was analyzed using generalized linear mixed‐effect model (“glmer” function provided by R package “lme4”) with a binomial error structure. The “ANOVA” function provided by R package “car” was used to estimate the significance of effect of explanatory variable. To examine the possibility that a changing trend over time actually ceased during the later stages of the experiment, we also performed analyses that progressively excluded earlier points in time until time effect became nonsignificant. General linear model was used for the dependence of population density and morphotype richness at certain points in time on population density at earlier sampling points and generalized linear model with a binomial error structure for ecological diversification state data. Comparison between populations from the end of the compensatory evolution experiment and the source populations in density and morphotype richness was carried out using paired‐*t* test and that in ecological diversification state (probability of containing two, vs. one, ecotypes) was analyzed using Exact test (R package “Exact”).

## 3 RESULTS

The antibiotic‐resistant morphotypes used to start the compensatory evolution experiment were from 42 source populations that had been founded with a single ancestral strain and evolved for six transfers in an antibiotic‐free environment. All the source populations consisted of two ecotypes (SM and WS) and showed relatively high morphotype richness (4.79 ± 0.0641), comparable to earlier observations with this model adaptive radiation (Fukami et al., 2007; Tan et al., 2013; Zhang et al., 2018).

**FIGURE 2:**
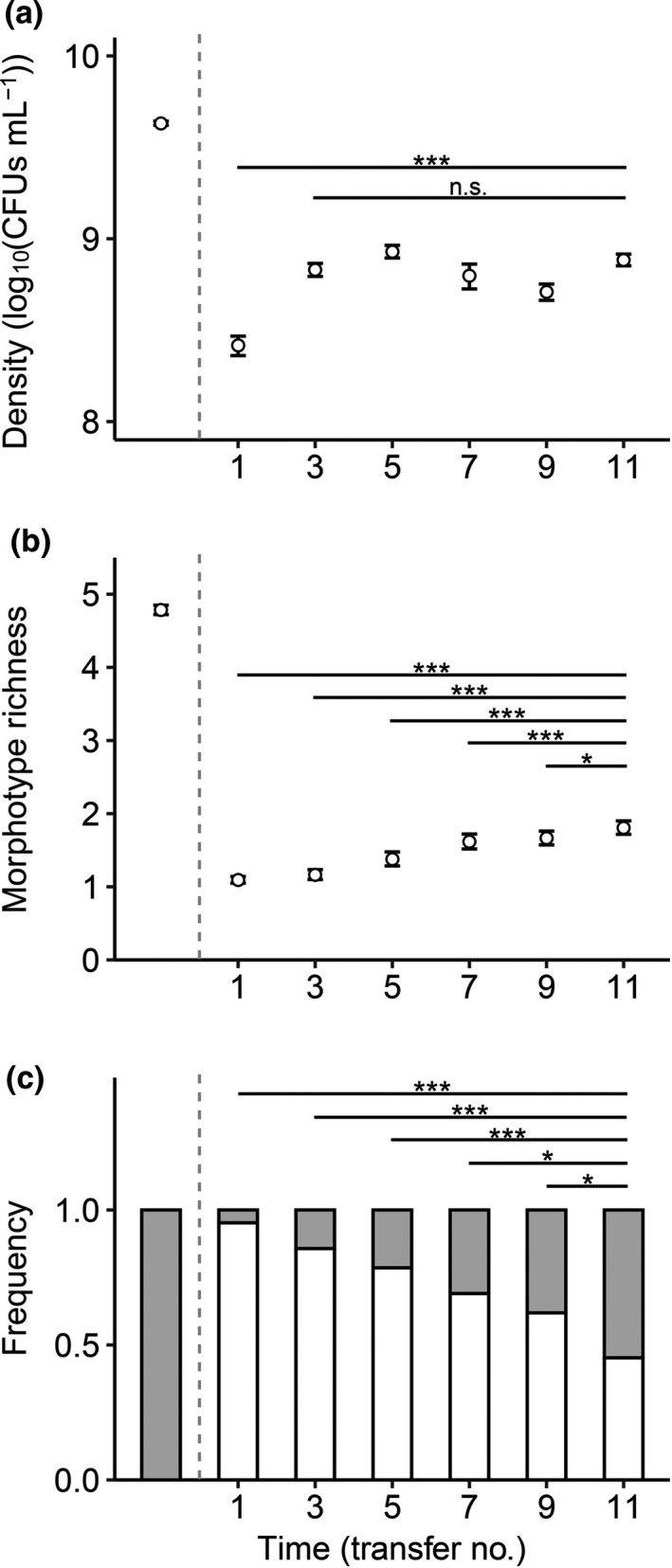
Population density (a), morphotype richness (b), and ecological diversification state (c) over time during compensatory evolution. Data in (a) and (b) show mean ± *SE* (*n* = 42); and those in (c) show the frequencies of ecologically diversified (2‐ecotype; filled bar) and nondiversified (1‐ecotype; open bar) populations. The leftmost data point in each panel represents the source populations grown in antibiotic‐free medium. Asterisks indicate significant time effect during certain periods of the compensatory evolution (*p < .05; **p < .01; ***p < .001)

Forty‐two populations seeded with single antibiotic‐resistant bacterial isolates underwent eleven transfers of compensatory evolution in antibiotic‐present nutrient medium. During this period, populations showed an increase in density over time; specifically, population density showed a rapid increase from transfer 1 to transfer 3, but ceased to increase since then (Figure 2a). There was a persistent increasing tend over time in morphotype richness throughout the experiment (Figure 2b). The chance that populations became diversified to contain two ecotypes also increased over time throughout the experiment (Figure 2c).

**FIGURE 3:**
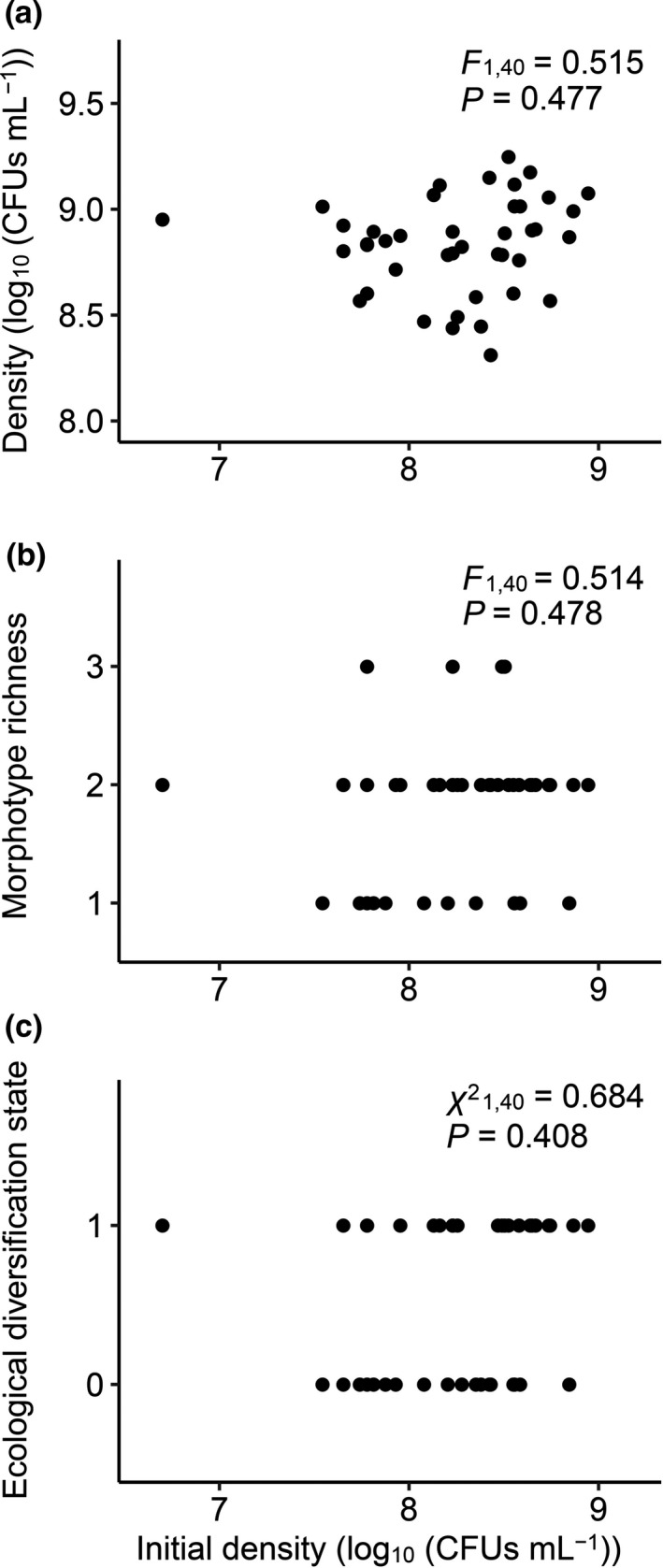
Relationship between population density at the beginning of compensatory evolution and population density (a), morphotype richness (b), and ecological diversification state (c) from the end of the experiment. Ecological diversification state is either 1 (containing two ecotypes) or 0 (containing only one ecotype)

**FIGURE 4:**
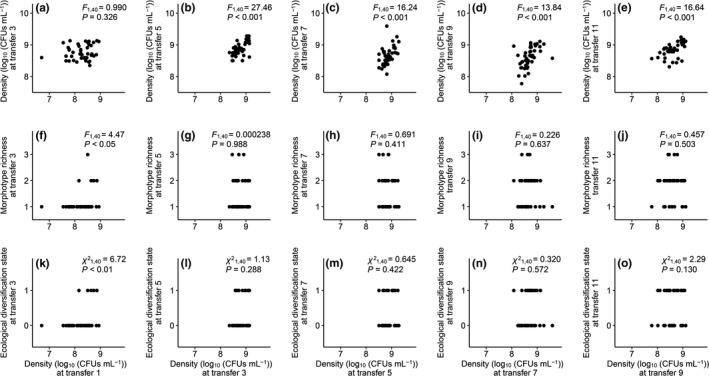
Relationship between population density (log_10_ (CFUs ml^−1^)) at certain points in time and population density (a–e), morphotype richness (f–j), and ecological diversification state (k–o) at immediately later sampling points in time

Population density at the beginning of the compensatory evolution experiment was not a significant predictor of final population density (Figure 3a), morphotype richness (Figure 3b), or ecological diversification state (Figure 3c). There was not a significant relationship between population density at transfer 1 and that at transfer 3 (Figure 4a); however, positive correlations were observed between consecutive sampling points in the later stages (Figure 4b–e). Populations with higher densities at transfer 1 showed greater morphotype richness at transfer 3 (Figure 4f), as well as a larger chance to be ecologically diversified (i.e., containing two, rather than one, ecotypes; Figure 4k). Such a relationship between population size and diversification was not observed during the later stages (Figure 4g–j,l–o).

Populations from the end of the compensatory evolution experiment grown in the presence of the antibiotic still had significantly lower population density compared with the source populations grown in antibiotic‐free medium (mean values of 7.68 × 10^8^ vs. 4.29 × 10^9^ ml^−1^; paired‐*t* test, *t*_41_ = −27.4, *p* < .001). Their morphotype richness was also lower than the source populations (mean values of 1.81 vs. 4.79; *t*_41_ = −25.8, *p* < .001); and the probability to diversify into two ecotypes was also smaller (23/42 vs. 42/42; exact test, *p* < .001).

## 4 DISCUSSION

Evolutionary rescue is not without costs. Here, we addressed how further evolution may compensate for the reduction in population size and ecological diversity associated with evolutionary rescue. In our experiment with *P*. *fluorescens* populations under the stress of antibiotic kanamycin, recovery of population size took place very rapidly, and the increase of diversity persisted longer (Figure 2). The paces of compensatory changes in population size and diversity may be explained by a contingency of ecological diversification on sufficiently large population size (Bailey et al., 2013; Buckling & Rainey, 2002; Kassen et al., 2000; Meyer & Kassen, 2007). Consistently, we found that populations with greater sizes were more likely to diversify at the very beginning of the compensatory evolution (transfer 1–3), though such a productivity–diversification relationship was observed during the later stages (Figure 4).

Explanation of our results from a fitness landscape perspective is tricky but could be insightful. Fitness landscapes are usually assumed being rugged (Cervera et al., 2016; Colegrave & Buckling, 2005; Cooper & Lenski, 2010; Melnyk & Kassen, 2011; Rozen et al., 2008); and it is expected that populations founded with better‐adapted genotypes are less likely to diversify. This prediction was supported by a previous study with *P*. *fluorescens* in a benign environment (Buckling et al., 2003), but not our experiment with an antibiotic environment. Population size (carrying capacity) of our experimental populations increased rapidly during the early stage of compensatory adaptation, and thus, it was likely an important fitness component at least during this early stage. Our observation that populations with larger sizes were more likely to diversify at the very beginning of the compensatory evolution is not consistent with the rugged fitness landscape view. Intriguingly, our results are instead consistent with a view of “holey” fitness landscapes that consist of connected networks of relatively well‐adapted genotypes (“ridges”) and aggregates of low‐fitness genotypes (“holes”) (Gavrilets, 1997, 1999; Gavrilets & Gravner, 1997). On such fitness landscapes, selection will be very effective in moving populations away from “holes”; however, evolutionary changes on “ridges” could be slow and drift‐like, and population diversification does not need cross “adaptive valleys.” There is a possibility that a “holey” fitness landscape emerged under our antibiotic treatment. The evolutionarily rescued populations thus showed a very rapid, short‐lived, increase in growth performance (moving from locations near “holes” to the “ridges”), and then slow changes in phenotype (moving on the “ridges). This explanation relies on a possibility that neutral coexistence may occur among different phenotypes in this experimental system, which has been previously demonstrated (Fukami et al., 2007; Zhang et al., 2009). This “holey” fitness landscape view may also explain the lack of a correlation between initial population size and final population size or diversity in our experiment (Figure 3).

Compensatory adaptation was far from being sufficient in our experiment. Densities of populations from the end of the compensatory evolution experiment were only ~18% of the source populations grown in the antibiotic‐free environment. Insurmountable genetic constraints may have existed for further improvement of growth performance. Known resistance mechanisms to the antibiotic kanamycin include synthesis of aminoglycoside modifying enzymes and changes of conformation of targets, 30S subunit bacterial ribosome (Melnyk et al., 2014; Shaw et al., 1993); and those may hamper normal physiological activities and lead to lower resource use efficiency. It would be interesting for future studies to reveal which particular physiological functions are associated with fitness costs that are difficult to overcome by compensatory adaptation. The recovery of diversity in our bacterial populations was of slow paces and persisted throughout our compensatory adaptation experiment which lasted for eleven transfers (note that adaptive radiation of the wild‐type *P*. *fluorescens* SBW25 in benign environments usually takes only two or three transfers; (Buckling & Rainey, 2002; Travisano & Rainey, 2000; Zhang et al., 2009)). While it is unclear whether diversity will recover to a level comparable to the source populations if the experiment has run for a much longer period, the slow pace of diversity increase suggests that environmental stress or fixation of resistance mutations have affected the accessibility of mutations responsible for niche shift, or the strength of diversifying selection. The slow recovery of within‐population diversity after evolutionary rescue implies that multiple, successive, environmental deterioration events, if existing, could be particularly harmful for population persistence and adaptation. Therefore, the promise of rapid evolution for the maintenance of biodiversity and ecosystem functioning under environmental deterioration should be considered with caution.

Our findings may also provide insights for ecosystem restoration in degraded environments. Reintroducing propagules of organisms into damaged habitats is a frequently used strategy in ecosystem restoration (Holl & Aide, 2011; Moreno‐Mateos et al., 2020). These organism propagules, however, are unlikely well‐adapted to the new environments; and the founding populations usually have low diversity. Therefore, adaptation subsequent to re‐introduction is akin to compensatory evolution following evolutionary rescue. The practice to introduce only organism varieties with the best growth performance in the degraded environments (Aschenbach, 2006; Li et al., 2019) may not guarantee desirable effects in the long run (Eizaguirre & Baltazar‐Soares, 2014; Hughes et al., 2008). This is because varieties with the best initial growth performance may not necessarily be those with larger evolutionary potential for fitness increase or diversification (as suggested by our results). Reintroduced populations in restoration programs, if only maintaining small population sizes and low genetic diversity, will be more likely to go extinct when faced with further, new, environmental stresses (Agashe et al., 2011; Carlson et al., 2014; Ramsayer et al., 2013). The potential for future population adaptation and diversity recovery may be enhanced instead by including more varieties including those with poor initial growth performance.

## CONFLICT OF INTEREST

None declared.

## AUTHOR CONTRIBUTIONS

**Dong‐Hao Zhou:** Conceptualization (supporting); data curation (equal); formal analysis (equal); funding acquisition (supporting); investigation (lead); methodology (equal); project administration (supporting); resources (equal); software (equal); supervision (equal); validation (equal); visualization (equal); writing‐original draft (equal); writing‐review & editing (equal). **Quan‐Guo Zhang:** Conceptualization (lead); data curation (equal); formal analysis (equal); funding acquisition (lead); investigation (supporting); methodology (equal); project administration (equal); resources (equal); software (equal); supervision (lead); validation (equal); visualization (equal); writing‐original draft (equal); writing‐review & editing (equal).

## Supporting information

Supplementary Material

## Data Availability

Data are available at Dryad: https://doi.org/10.5061/dryad.3n5tb2rhq.
