# Supplementary material for: Compensatory adaptation and diversification subsequent to evolutionary rescue in a model adaptive radiation
Source: Ecol Evol. 2021 Jun 16;11(14):9689–96. doi: 10.1002/ece3.7792 (PMC8293784; doi:10.1002/ece3.7792)
Supplement: Supplementary file 1 — Supplementary Material [file ECE3-11-9689-s001.docx]

**Compensatory adaptation and diversification subsequent to evolutionary rescue in a model adaptive radiation**

**Supporting information**

**Table S1. Bacterial morphotypes identified in the present study.**

| **Identity** | **morphological** **characteristics** |
| --- | --- |
| Large SM | Circular, normal-sized, SM |
| Small SM | Circular, small-sized, SM |
| Large WS | Irregular, large-sized, WS |
| Small WS | Irregular, small-sized, WS |
| SM-like WS | Irregular WS with SM-like parts |
| Wheel-like WS | Circular, wheel-like, WS |
| Round WS | Circular WS, not wheel-like |
